# Resilience and Mental Well-Being During the COVID-19 Pandemic: Serial Mediation by Persistent Thinking and Anxiety About Coronavirus

**DOI:** 10.3389/fpsyt.2021.810274

**Published:** 2022-01-27

**Authors:** Sebastian B. Skalski, Karol Konaszewski, Arndt Büssing, Janusz Surzykiewicz

**Affiliations:** ^1^Institute of Psychology, Polish Academy of Sciences, Warsaw, Poland; ^2^Faculty of Education, University of Białystok, Białystok, Poland; ^3^Professorship Quality of Life, Spirituality and Coping, Faculty of Health, Witten/Herdecke University, Witten, Germany; ^4^Faculty of Philosophy and Education, Catholic University of Eichstaett-Ingolstadt, Eichstaett, Germany; ^5^Cardinal Stefan Wyszynski University in Warsaw, Warsaw, Poland

**Keywords:** resilience, well-being, COVID-19, anxiety, persistent thinking

## Abstract

Reports to date indicate that the COVID-19 pandemic outbreak has negatively impacted mental health in the general population. On the other hand, positive associations of mental resilience and well-being have been widely demonstrated. The objective of this study was to assess the links between resilience (Brief Resilience Scale), persistent thinking about COVID-19 (Obsession with COVID-19 Scale), coronavirus anxiety (Coronavirus Anxiety Scale), and well-being (World Health Organization's 5-item Well-being Index) using serial mediation. The study considered online survey data from 1,547 Poles aged 18–78 (62% of whom were women). Bootstrap sampling analysis revealed that persistent thinking about COVID-19 (M1) and coronavirus anxiety (M2) partially mediate the relationship between resilience and well-being. The results of this study indicate that persistent thinking may be dysfunctional for mental health, as it inflates pandemic anxiety and disrupts well-being. Moreover, practitioners should focus on interventions enhancing resilience in order to reduce negative mental effects during the spread of a pandemic infectious disease.

## Introduction

The outbreak of the COVID-19 pandemic has had a negative impact on the daily functioning of individuals, affecting their mood, sense of well-being and, consequently, their overall mental health. Numerous publications on the consequences of COVID-19 have demonstrated symptoms of post-traumatic stress disorder (PTSD), chronic stress, sleep disorders, prevalence of suicidal thoughts, complicated grief, social anxiety, or substance abuse among medical personnel, convalescents, and in the general population (1–5). According to Lee (6), the nature of the pandemic has led to constant updating of the media news on COVID-19, which may have elevated the experienced level of anxiety. Excessive consumption of information often accompanied by conflicting or unverified messages about the spread of infectious disease is believed to lead to dysfunctional thought processes (persistent thinking) and is devastating to the mental health of individuals (7). In the face of COVID-19, persistent dysfunctional thinking may have manifested as the dissipation of epidemic threats in the form of intrusive personal memories and transmitted via electronic media, tormenting dreams with epidemic-related content, repetitive dissociative reactions associated with a sense of the unreality of the threat, and a persistent sense of hurt and suffering (modeled on the psychological effects of the Ebola epidemic in America) (8, 9).

Persistent and disturbed thinking (a.k.a. obsessions) is recognized as a psychopathological symptom denoting the presence of persistently recurring thoughts, ideas, or impulses that impede daily functioning (10). Obsessions occur against the individual's will (they are ego-dystonic). They disrupt thinking and prevent from focusing one's attention on something else (11). Persistent thinking causes the build-up of emotional tension (e.g., feeling anxious, worried, fearful). In addition, it can lead to the performance of so-called intrusive activities (compulsions) that co-occur with obsessions (12). As per the ICD-11 and DSM-5 classifications, obsessions are among the symptoms typical of obsessive-compulsive disorder (13, 14). However, it should be clearly underlined that not all (justified) fears and worries related to the pandemic are indicators of (pathological) obsessive thinking.

It is commonly believed that cognitive distortion arising from interpretations based on dysfunctional assumptions and core beliefs are the source of persistent thinking (15). Cognitive distortions are ways of thinking that negatively distort the way we see the world, ourselves and others. According to Beck et al. (16), cognitive distortions cause one to absorb the threat and underestimate the ability to cope with it, which ultimately leads to pathological anxiety. These findings were extended in later theories describing aspects of mind and behavior. As per LeDoux (17), emotions (like vegetative and behavioral responses) are the product of complex, evolutionarily produced brain mechanisms designed to defend against danger. The author assumes that a person experiencing physiological stimulation has to evaluate, name, and then understand the emotion being felt, which they do most often based on situational context and previous experiences (17). In the process of “becoming aware” of one's emotions, an individual may make numerous errors (i.e., cognitive distortions). Both Beck et al. (16) and LeDoux (17) assume that emotions are the result of cognitive interpretations of situations, which is in line with cognitive concepts that focus on restructuring the mental representation of emotional arousal (18). This arousal always remains dependent on an individual's interpretation of the social context. In other words, subjectively experiencing external factors determines the quality, intensity, and persistence of internal emotional experiences and generates a tendency toward specific behavioral responses (19).

The occurrence of cognitive distortions manifested in the form of persistent and disturbed thinking in relation to COVID-19, as well as subsequent negative emotional experiences, have been demonstrated in numerous reports. It was estimated that in 2020 the phenomenon of persistent thinking about COVID-19 affected 12–20% of the general population, with the most common symptoms being intrusive beliefs about becoming infected with SARS-CoV-2, as well as repeated thoughts about having to undergo coronavirus testing despite the absence of infection symptoms (20–24). Researchers have also demonstrated the effect of persistent thinking on the severity of coronavirus anxiety (25, 26), which is sometimes interchangeably referred to as *coronaphobia* (27).

The occurrence of negative effects of pandemics and subsequently associated social restrictions on individuals' mental health, such as cognitive distortions and subsequent negative emotional experiences, requires researchers and practitioners to develop intervention methods to safeguard well-being during the spread of infectious diseases. Studies to date have shown that psychological resilience positively stabilizes mental health during the COVID-19 pandemic (28). Strong associations of resilience with well-being, quality of life, and life satisfaction have been widely reported in studies (29). On the other hand, Labrague and Santos (30) showed that resilience was a significant (negative) predictor of COVID-19 anxiety, and that an intervention to increase personal resilience alleviated coronaphobia. Moreover, Skalski et al. (31) showed that fear of the spread of infectious disease mediated the relationship between resilience and PTSD symptoms among the general population. Meanwhile, Surzykiewicz et al. (32) exhibited that the relationship of resilience and well-being, in addition to coronaphobia, was also mediated by persistent dysfunctional thinking about COVID-19. However, with respect to the indicated studies and assumptions, Beck et al. (16) and LeDoux (17), it appears that the link between resilience and coronavirus anxiety (with well-being as an outcome variable) may be further mediated by persistent thinking about COVID-19.

The basis of our study is a theoretical model of individual resilience that allows us to understand the role of potential mediators in the link between resilience and well-being (33). This conceptualization also provides a foundation for establishing determinants of psychological adjustment (e.g., anxiety, stress, depression). The theory by Rees et al. (33) includes several intrapersonal constructs that play a key role in the assessment of and response to stressors. According to this model, a person's resilience is significantly related to mental health outcomes. Individuals who score higher on measures of resilience simultaneously obtain higher results regarding measures of good psychological functioning. While this relationship is obvious, little attention has been paid in the literature to the role of potential mediators in the link between resilience and well-being. On the other hand, available reports generally agree that resilient individuals are less likely to report symptoms of distress and anxiety during traumatic events, thereby maintaining optimal levels of well-being (32). At the same time, stress, anxiety, fear, and depressive symptoms have been identified as key negative predictors of mental health (30). Additionally, fear of the spread of infectious disease has been defined as a marker of mental health during the pandemic and is used to screen for psychological functioning during COVID-19 (31). Thus, it appears that the relationship of resilience and well-being may be explained by persistent thinking and fear of the occurrence of infectious disease.

One such modifiable construct that enhances mental health may be resilience. In our study, resilience is defined as the ability of individuals to resist or “bounce back from adversity.” Understanding resilience as “the ability to bounce back” or recover from stress can be important for assessing the positive and negative indicators of the functioning of individuals and, in particular, for designing activities aimed at shaping resilience when understood in terms of an ability. Furthermore, this ability may be extremely important for people who are already sick or have to deal with constant stress related to mental health (34). It is reasonable to assume that promoting well-being and resilience is relevant in the prevention of psychological burden and clinical symptoms.

### Objective of the Study

As per the above literature review, resilience may helps to safeguard mental health during the spread of a pandemic infectious disease. However, this is also depending on the course of the disease, as some resources may exhaust in long periods of burden or stress, as can be seen during the different phases of the COVID-19 pandemic. On the other hand, the media plays a vital role in disseminating information during a pandemic, and constant exposure to COVID-19-related information in both traditional and social media leads to persistent and distorted thinking. Persistent thinking, meanwhile, causes emotional tension to build up, i.e., it elevates anxiety. Consequently, one's mental well-being may be impaired, not least because of the immediate negative effects of the pandemic. The authors of this article consider whether the relationship between resilience and well-being may be serially mediated by persistent dysfunctional thinking about COVID-19 and coronavirus anxiety. Similar studies have not been conducted before. The data obtained will allow for a better understanding of the links between the described variables and will also contribute to the development of effective intervention methods.

For the purpose of this study, the following working hypothesis has been adopted: the link between resilience and well-being may be serially mediated by persistent thinking about COVID-19 (M1) and the coronavirus anxiety (M2). Although the adopted model is strongly backed by cognitive-behavioral theories, at the same time some researchers argue that anxiety may be linked with the feeling of hopelessness and may reinforce persistent thinking (25). In light of this, we have decided to assess an alternative model as well, in which we reversed the order of the mediators.

## Methods

### Participants and Procedure

The cross-sectional online survey was conducted with the approval of the university ethics committee. Data were collected in Poland in spring 2021 (during the so-called third wave of COVID-19) among adults in the general population. Participation in the study was anonymous and required consent. The invitation to participate was made available through national media (websites) and social networking sites (e.g. Facebook). The recruitment did not require additional criteria to be met. Data from the online surveys were collected on the Microsoft Forms platform and then exported to a spreadsheet. The study involved 1,547 individuals aged 18–78 (*M* = 44.67, *SD* = 12.41), including 62% females. Of the participants, 4% were actively infected with SARS-CoV-2, while 11% were convalescents. None of the participants worked directly on COVID-19 outbreak control (e.g. in a hospital). The study procedure consisted of filling in psychological questionnaires measuring resilience, persistent thinking about COVID-19, coronavirus anxiety and well-being. The test lasted about 15 min.

### Measures

The Brief Resilience Scale (BRS) was used to measure mental resilience (34). The single-factor scale consists of six self-descriptive statements. The participants rate each of them on a 5-point Likert scale, from 1 = “*I strongly disagree”* to 5 = “*I strongly agree.”* The Polish version of the BRS is characterized by good psychometric properties (Cronbach's α = 0.88) (35). Sample items: “*I tend to bounce back quickly after hard times”*; “*I tend to take a long time to get over set-backs in my life.”*

The Obsession with COVID-19 Scale (OCS) was used to measure persistent thinking about COVID-19 (6). The single-factor scale consists of four statements relating to experiences over the past two weeks. The participants express their attitude toward each of the statements on a 5-point Likert scale (0 = “*Not at all”* to 4 = “*Almost everyday”*). The Polish version of the OCS is characterized by good psychometric properties (Cronbach's α = 0.82) (25). Sample items: “*I had disturbing thoughts that I may have caught the coronavirus”*; “*I felt nauseous or had stomach problems when I thought about or was exposed to information about the coronavirus.”*

The Coronavirus Anxiety Scale (CAS) was used to measure coronavirus anxiety (36). The single-factor scale consists of five statements relating to experiences over the past two weeks. The response manner is coherent with OCS. The Polish version of the CAS is characterized by good psychometric properties (Cronbach's α = 0.86) (32). Sample items: “*I lost interest in eating when I thought about or was exposed to information about the coronavirus”*; “*I felt nauseous or had stomach problems when I thought about or was exposed to information about the coronavirus.”*

The World Health Organization's 5-item Well-being Index (WHO-5) was used to measure well-being (37). The scale consists of five self-descriptive statements. The participants express their attitude toward each of the statements on a 6-point Likert scale (in relation to the past two weeks), from 0 = “*At no time”* to 5 = “*All of the time.”* The Polish version of the WHO-5 is characterized by good psychometric properties (Cronbach's α = 0.87) (38). Sample items: “*I have felt cheerful in good spirits”*; “*I have felt calm and relaxed.”*

### Statistical Analyses

All analyses were performed using the SPSS software rev. 26 and the PROCESS 4.0 plug-in for mediation effects analysis. Assessment of normality was performed using the Kolmogorov-Smirnov test, whereas homoscedasticity of variance was assessed using Levene's test. The data allowed for applying parametric tests. The evaluation of relationships between variables was performed using Pearson's *r* correlation analysis. The analysis of the serial mediation effect (model 6) was conducted using the bootstrap method. The effect size was assessed on the basis of *R*^2^. The Bootstrap analysis sample size was 5,000 and the mediation effect test is significant when it does not contain zero under the 95% confidence interval. The significance level was determined at *p* ≤ 0.050.

## Results

Descriptive statistics and correlations are presented in Table 1. Resilience correlated moderately positive with well-being, and marginally negative with persistent thinking about COVID-19 and coronavirus anxiety. This persistent thinking was marginally only related to low well-being, while coronavirus anxiety was at least weakly negatively related to well-being. Active SARS-CoV-2 infection (0 = “*No active infection”*, 1 = “*Active infection”*) was linked to higher rate of persistent thinking about COVID-19 (*r* = 0.27; *p* < 0.001) and coronavirus anxiety (*r* = 0.29; *p* < 0.001), and was negatively but irrelevantly correlated with resilience (*r* = −0.09; *p* < 0.001) and marginally negative with well-being (*r* = −0.11; *p* < 0.001). Moreover, a significant but irrelevant positive association was observed between age and resilience (*r* = 0.09; *p* < 0.001). Other sociodemographic data were not statistically significantly associated with the results. Prior to performing a mediation analysis, in relation to the occurrence of a correlation between predictors, a collinearity test was performed. Each time, the Variance Inflation Factor (*VIF*) rated between 1.04 and 1.33, with the tolerance indicators returning a value between 0.75 and 0.96, which indicates the lack of collinearity between independent variables.

**Table 1:** Descriptive statistics and correlations (*N* = 1,547).

	**M (SD)**	**1**.	**2**.	**3**.
1. Resilience	18.89 (5.31)	1		
2. Persistent thinking about COVID-19	1.06 (2.53)	−0.16^***^	1	
3. Coronavirus anxiety	2.64 (2.92)	−0.17^***^	0.49^***^	1
4. Well-being	14.07 (5.65)	0.37^***^	−0.19^***^	−0.28^***^

*** *p < 0.001*.

Bootstrap sampling analysis showed statistically significant serial mediation. The model assessed the link between resilience (*X*), persistent thinking about COVID-19 (*M1*), coronavirus anxiety (*M2*) and well-being (*Y*). As a result of the analysis, a positive direct relationship between resilience and well-being was observed (total effect; *B* = 0.392; *SE* = 0.025; *95% CI* = 0.343, 0.441; *R*^2^ = 0.14). After including mediators of persistent thinking about COVID-19 and coronavirus anxiety in the analysis, the relationship coefficient decreased but was still statistically significant (direct effect; *B* = 0.349; *SE* = 0.025; *95% CI* = 0.300, 0.397; *R*^2^ for the entire model = 0.19) Resilience also proved to be a negative yet less relevant predictor of persistent thinking about COVID-19 (*B* = −0.078; *SE* = 0.012; *95% CI* = −0.102, −0.055; *R*^2^ = 0.03) and coronavirus anxiety (*B* = −0.091; *SE* = 0.138; *95% CI* = −0.118, −0.064; *R*^2^ = 0.03).

**Figure 1:**
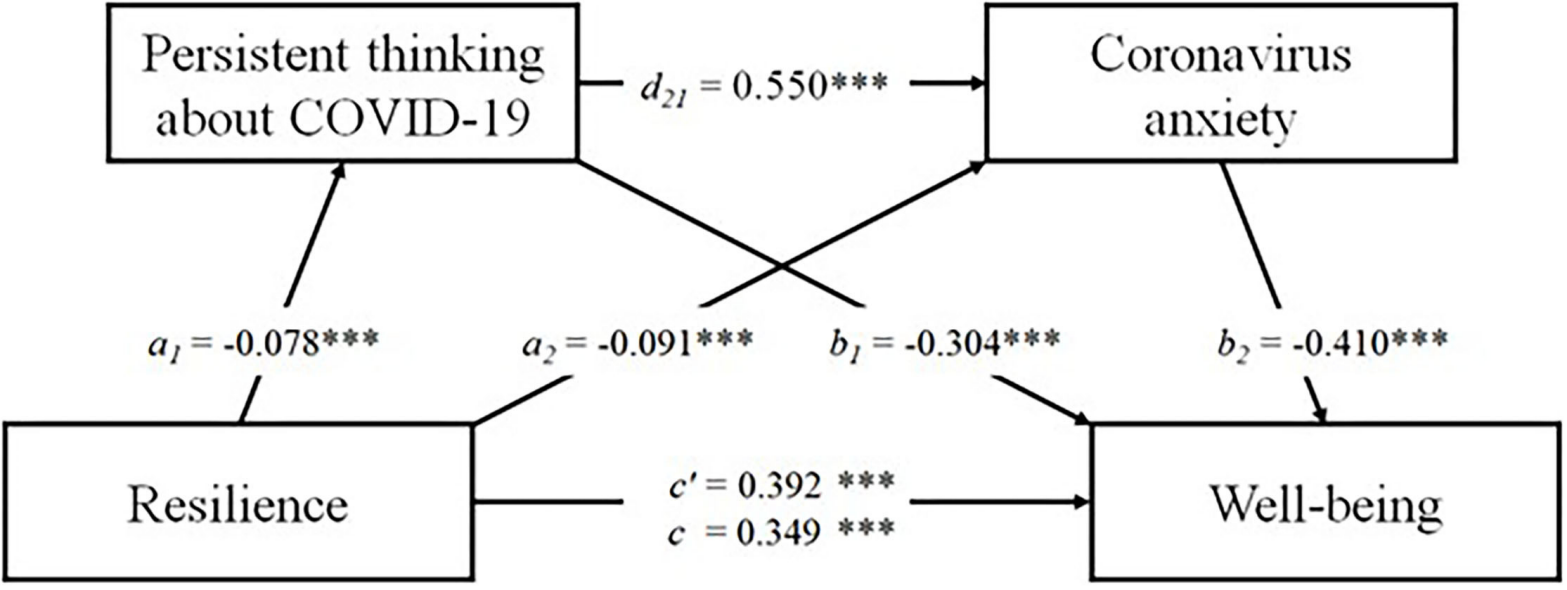
The result of serial multiple mediational model, ***p < 0.001. Values shown are unstandardized coefficients.

The analyses revealed a significant indirect effect of resilience on well-being through persistent thinking about COVID-19 (*B* = 0.024; *SE* = 0.006; *95% CI* = 0.014, 0.036; *R*^2^ = 0.16). The indirect effect of resilience on well-being via coronavirus anxiety was also found to be significant (*B* = 0.040; *SE* = 0.008; *95% CI* = 0.027, 0.056; *R*^2^ = 0.18). Finally, the study assessed the indirect impact of resilience on well-being through both persistent thinking about COVID-19 and coronavirus anxiety. The relationship was significant with a point estimate of 0.017 (testing serial multiple mediation; *SE* = 0.004, 95%*CI* = 0.010, 0.024; *R*^2^ = 0.28). A visualization of the mediation model is presented in Figure 1. The alternative model, where the mediator order has been reversed (M1: coronavirus anxiety; M2: persistent thinking about COVID-19) was not significant with a point estimate of 0.003 (*SE* = 0.002, 95%CI = −0.002, 0.008; *R*^2^ = 0.19).

## Discussion

The objective of this study was to assess the links between resilience, persistent thinking about COVID-19, coronavirus anxiety and well-being. Our results suggest that persistent thinking and pandemic-related anxiety can mediate the relationship between resilience and well-being. The data obtained correspond with previous reports that indicate an increase in negative psychological consequences during COVID-19 (1–5). According to researchers, well-being may be compromised during a pandemic by direct and indirect trauma, potential risk perception, disruption of daily routines, reduced social support, and feelings about perceived loss of control (39). Furthermore, our data suggest that resilience may increase the level of well-being during the spread of an infectious disease, which also supports previous empirical findings in this area (28, 31, 32).

We were the first to show that the relationship between resilience and well-being may be serially mediated by persistent thinking about COVID-19 and coronavirus anxiety (partial mediation). As per the data obtained, resilience may protect well-being by reducing persistent thinking about COVID-19, which promotes lower levels of coronavirus anxiety. However, this mediation model is depending on the included groups; some may be more at risk and have stronger fears and thus lower well-being, while others are less afraid to be infected (regardless of whether this assumption is justified or not) and may have thus better well-being. Our results indirectly correspond with the study by Satici et al. (40), according to which the relation between intolerance of uncertainty and well-being was serially mediated by persistent thinking and fear of COVID-19. Meanwhile, Ciesla and Roberts (41) showed that the relationship between persistent thinking and depressive mood is mediated by negative cognitions. Based on the above empirical findings, we hypothesize that persistent dysfunctional thinking elevates negative cognition and mood states and decreases well-being. On the other hand, resilience may promote the reduction of persistent thinking, which may reduce further negative mental effects in traumatic and uncertain situations. Furthermore, the alternative model with the reversed mediator order proved to be insignificant, which also suggests that persistent thinking about COVID-19 should be best perceived as stimuli that can lead to coronavirus anxiety, yet they should not be treated as cognitive reactions as a result of psychological stress and the feeling of danger.

In our study, individuals infected with SARS-CoV-2 showed higher rates of persistent thinking and anxiety in relation to COVID-19, and lower rates of resilience and well-being. This seems understandable, as coronavirus infection is associated with loss of health resources and requires quarantine and thus social isolation. Depending on the course of the infection (either mild or with hospitalization), their fears and worries might be justified. However, as only 4% were actively infected with SARS-CoV-2, while 11% recovered, most of the sample (85%) may not have concrete COVID-19 and their rather low scores of anxiety and obsessive thinking could also be a matter of carelessness - and thus their wellbeing might be higher as compared to the others. Active infection also increases exposure to COVID-19 information (e.g., health reports from your doctor). These observations correspond to previous reports (25, 32, 33). In addition, it should be noted that researchers believe the link between exposure to stress and the level of resilience may be moderated by individual differences, including the nature and intensity of psychological and physiological reactions to stress and the frequency of exposure to stress, which allows for further potential explanation of the negative association between active coronavirus infection and resilience (42). In this sample of Polish participants, age was not relevantly related to a higher resilience index. This observation corresponds with previous reports (35). It is believed that resilience may increase together with the accumulating experience of coping with challenging events and circumstances through life (43); however, this was not substantially approved in this study. It should be noted that the lack of statistically significant associations between age and gender vs. the dependent variable precludes consideration of these demographic variables as covariates.

Despite its strengths, our report is associated with some limitations. First of all, the study was correlational and self-descriptive. Therefore, cause and effect should not be conclusively inferred. In future research, it seems interesting to collect data from an experiment, diary method, or longitudinal study to obtain more reliable data. In addition, our study was conducted among the general population with quite low concrete experience with CVID-19 infection. Future studies should also enroll persons at risk (i.e., hospital staff) and should compare persons who ignore protection recommendations with those who strictly adhere to it, assuming the first may have low Corona anxiety and are not constantly thinking about COVID-19, while the others may be more aware of the risks. Hence, caution should be exercised when applying the results of this report to a clinical population and other countries. Thirdly, we only controlled baseline demographic variables (age and sex) in the study. The inclusion of detailed socio-demographic data (e.g., socioeconomic background, partner status, strictness of religious faith) would have allowed us to determine the impact of these side variables on the constructs evaluated. The obtained data suggest that persistent dsysfunctional thinking about an infectious disease allows for predicting well-being to some degree by elevating pandemic anxiety. In the context of future studies, it would seem interesting to explore other potential mediators for this relationship, such as existential loneliness and meaninglessness.

This is the first study to assess the link between resilience, persistent obsessive thinking, pandemic anxiety, and well-being using serial mediation. Our data indicated that persistent obsessive thinking about the spread of an infectious disease is dysfunctional for mental health because it inflates pandemic anxiety and disrupts well-being. On the other hand, resilience promotes the reduction of persistent thinking and anxiety, which in turn helps to secure well-being in traumatic and uncertain situations. Our results have an application value. They can be used in the development of prevention programs and therapeutic interventions. According to the data obtained, interventions that increase resilience may promote elevated levels of well-being during the spread of a pandemic infectious disease.

## Data Availability Statement

The raw data supporting the conclusions of this article will be made available by the authors, without undue reservation.

## Ethics Statement

The studies involving human participants were reviewed and approved by the Ethics Committee of the Witten/Herdecke University. The patients/participants provided their written informed consent to participate in this study.

## Author Contributions

The first draft of the manuscript was written by SS and KK. SS, KK, and JS conceived and designed the study, analyzed the data, wrote the manuscript, and interpreted the results. SS, KK, AB, and JS drafted the manuscript and read and approved the final version. KK and JS performed the study. All authors contributed to the article and approved the submitted version.

## Conflict of Interest

The authors declare that the research was conducted in the absence of any commercial or financial relationships that could be construed as a potential conflict of interest.

## Publisher's Note

All claims expressed in this article are solely those of the authors and do not necessarily represent those of their affiliated organizations, or those of the publisher, the editors and the reviewers. Any product that may be evaluated in this article, or claim that may be made by its manufacturer, is not guaranteed or endorsed by the publisher.
